# Predictive biomarkers of immunotherapy response with pharmacological applications in solid tumors

**DOI:** 10.1038/s41401-023-01079-6

**Published:** 2023-04-13

**Authors:** Szonja Anna Kovács, János Tibor Fekete, Balázs Győrffy

**Affiliations:** 1 Department of Bioinformatics, Semmelweis University, Tűzoltó utca 7-9, 1094 Budapest, Hungary; 2 Doctoral School of Pathological Sciences, Semmelweis University, Üllői út 26, 1085 Budapest, Hungary; 3 National Laboratory for Drug Research and Development, Magyar tudósok körútja 2 1117, Budapest, Hungary; 4 Research Centre for Natural Sciences, Oncology Biomarker Research Group, Institute of Enzymology, Eötvös Loránd Research Network, Magyar Tudósok körútja 2, 1117 Budapest, Hungary; 5 Department of Pediatrics, Semmelweis University, Tűzoltó utca 7-9, 1094 Budapest, Hungary

**Keywords:** immunotherapy, immune checkpoint inhibitors, gene expression, ROC curve, druggable genes, drug resistance

## Abstract

Immune-checkpoint inhibitors show promising effects in the treatment of multiple tumor types. Biomarkers are biological indicators used to select patients for a systemic anticancer treatment, but there are only a few clinically useful biomarkers such as PD-L1 expression and tumor mutational burden, which can be used to predict immunotherapy response. In this study, we established a database consisting of both gene expression and clinical data to identify biomarkers of response to anti-PD-1, anti-PD-L1, and anti-CTLA-4 immunotherapies. A GEO screening was executed to identify datasets with simultaneously available clinical response and transcriptomic data regardless of cancer type. The screening was restricted to the studies involving administration of anti-PD-1 (nivolumab, pembrolizumab), anti-PD-L1 (atezolizumab, durvalumab) or anti-CTLA-4 (ipilimumab) agents. Receiver operating characteristic (ROC) analysis and Mann-Whitney test were executed across all genes to identify features related to therapy response. The database consisted of 1434 tumor tissue samples from 19 datasets with esophageal, gastric, head and neck, lung, and urothelial cancers, plus melanoma. The strongest druggable gene candidates linked to anti-PD-1 resistance were *SPIN1* (AUC = 0.682, *P* = 9.1E-12), *SRC* (AUC = 0.667, *P* = 5.9E-10), *SETD7* (AUC = 0.663, *P* = 1.0E-09), *FGFR3* (AUC = 0.657, *P* = 3.7E-09), *YAP1* (AUC = 0.655, *P* = 6.0E-09), *TEAD3* (AUC = 0.649, *P* = 4.1E-08) and *BCL2* (AUC = 0.634, *P* = 9.7E-08). In the anti-CTLA-4 treatment cohort, *BLCAP* (AUC = 0.735, *P* = 2.1E-06) was the most promising gene candidate. No therapeutically relevant target was found to be predictive in the anti-PD-L1 cohort. In the anti-PD-1 group, we were able to confirm the significant correlation with survival for the mismatch-repair genes *MLH1* and *MSH6*. A web platform for further analysis and validation of new biomarker candidates was set up and available at https://www.rocplot.com/immune. In summary, a database and a web platform were established to investigate biomarkers of immunotherapy response in a large cohort of solid tumor samples. Our results could help to identify new patient cohorts eligible for immunotherapy.

## Introduction

Immune-checkpoint inhibitors (ICIs) were introduced for the treatment of solid and hematological malignancies with outstanding results in the last decade [1]. There are three groups of ICIs. The first group consists of ICIs inhibiting cytotoxic T lymphocyte-associated protein 4 (CTLA-4) on T cells [2, 3], the second one is related to programmed cell death 1 (PD-1) receptor on lymphocytes [4], and the third group is linked to programmed cell death ligand 1 (PD-L1) on tumor cells. Physiologically, PD-1 is expressed on several immune cells (e.g., lymphocytes, natural killer cells), and PD-L1 is present on almost all somatic cells (e.g., hematopoietic cells or tumor cells). In tumors, blockade of the PD-1/PD-L1 axis results in the survival of the malignant cells [5].

The first ICI approved by the U.S. Food and Drug Administration (FDA) for treating metastatic melanoma was ipilimumab, a fully human monoclonal antibody against CTLA-4 [6]. Ipilimumab was shown to be effective in an extremely wide range of advanced cancers including melanoma [7, 8], renal cell carcinoma (RCC) [9, 10], mismatch repair deficient (dMMR) or microsatellite instability-high (MSI-H) colorectal carcinoma [11], hepatocellular carcinoma (HCC) [12], pleural mesothelioma [13], and non-small-cell lung cancer (NSCLC) [14]. Tremelimumab, another fully human monoclonal antibody against CTLA-4, has been investigated in multiple solid tumors with mixed results [15]. Tremelimumab has not yet received an FDA-approval so far.

Nivolumab was the first approved monoclonal antibody targeting PD-1 and can be administered in advanced melanoma, Hodgkin’s lymphoma, HCC, NSCLC and SCLC, RCC, head and neck cancer, urothelial carcinoma, CRC [5], gastric, or esophageal adenocarcinoma [16], and malignant pleural mesothelioma [13]. Pembrolizumab, a humanized monoclonal antibody against PD-1 has been approved either as a monotherapy or as a combination therapy for the treatment of recurrent or metastatic melanoma [17], NSCLC, head and neck squamous cell cancer (HNSCC), gastric/gastroesophageal junction adenocarcinoma, lymphoma, urothelial cancer, cervical cancer, Merkel cell carcinoma, RCC [5], triple-negative breast cancer (TNBC) [18], cutaneous squamous cell carcinoma [19, 20], endometrial cancer [21], and HCC [22]. Pembrolizumab can be administered to patients regardless of their age and tumor type in case their tumor is MSI-H or dMMR [23]. Recently, dostarlimab, a humanized anti-PD-1 monoclonal antibody, has also received an FDA-approval for recurrent or advanced MSI-H/dMMR endometrial cancer and other solid tumors [24, 25].

PD-L1-blocking antibodies started with avelumab in 2015 for metastatic Merkel cell carcinoma, and then it continued with locally advanced or metastatic urothelial carcinoma (mUC), and advanced RCC. Durvalumab, another human monoclonal antibody was accepted for metastatic urothelial bladder cancer, urothelial carcinoma, NSCLC, and SCLC [5, 26]. Atezolizumab, a third PD-L1 monoclonal antibody, is also accepted for locally advanced or mUC, metastatic NSCLC, SCLC, TNBC, HCC, and melanoma [5, 27, 28].

Biomarkers are biological indicators that can be used to select patients for a systemic anticancer treatment like immunotherapy. A major limitation of the widespread use of immunotherapies is the fact that there are only a few clinically useful biomarkers capable to predict therapy response. A study [29] found that tumor mutational burden (TMB), and PD-L1 expression can predict response to pembrolizumab in a huge variety of cancers (melanoma, bladder cancer, breast cancer, CRC, HNSCC, and SCLC). The correlation between PD-L1 expression and MSI and response to pembrolizumab was also investigated in gastric cancer [30]. Cluster of Differentiation 8 positive (CD8^+^) T cell phenotype and TMB were associated with enhanced response to atezolizumab in mUC [31]. Findings from other studies highlighted the central role of the tumor microenvironment (TME) in nivolumab [32], pembrolizumab [33], and anti-CTLA-4 response [34]. The importance of both innate, and adaptive immune systems was emphasized in connection with anti-PD-1 response in NSCLC [35, 36], melanoma [36, 37], and HNSCC [36]. Meanwhile, in recent years, the tumors of several ICI-treated patient cohorts were investigated with transcriptomic technologies. The simultaneous analysis of the entire transcriptome makes it possible to identify new genes capable to serve as biomarkers of response and to validate previously suggested biomarker candidates.

Here, our goal was to expose and integrate available transcriptomic datasets of ICI-treated tumors to establish a framework enabling an integrated analysis of genes related to treatment sensitivity or resistance. Uncovering robust genes with increased expression in treatment-resistant tumors could offer the opportunity to develop a targeted therapy to augment the effects of immune-checkpoint inhibitors.

## Materials and methods

### ICI dataset screening

We screened the NCBI Gene Expression Omnibus (GEO) repository using the keywords “human [organism] AND (anti-PD-1 OR anti-PD-1 OR anti-PD-L1 OR anti-PD-L1 OR anti-CTLA-4 OR anti-CTLA-4)”, and “human [organism] AND (pembrolizumab OR nivolumab OR atezolizumab OR durvalumab OR avelumab OR cemiplimab OR ipilimumab OR camrelizumab OR cintilimab OR tislelizumab OR toripalimab)” on 10th Jan 2022. We omitted datasets with no available gene expression or clinical data, or with single-cell RNA-sequencing (scRNA-Seq), T or B cell receptor sequencing (TCR/BCR-Seq), non-mRNA-sequencing (e.g., whole-exome sequencing, non-coding RNA profiling, methylation profiling, protein array), studies of cell lines, stem cells, sorted peripheral blood mononuclear cells, studies in mice, studies without cancer, and GEO SuperSeries files. We also conducted a literature research to find additional studies. Our scope of investigation only included studies with simultaneously available clinical (response) and bulk-tissue gene expression data.

### Database setup

Gene expression data from all eligible datasets were combined into a single table, quantile normalized and scaled to 1000. For the clinical annotation, we categorized patients as responders or non-responders based on reported pathological response or survival time. Those patients were selected as responders who experienced progression-free survival (PFS) longer than 12 months or had a partial response (PR) or complete response (CR). Those who experienced less than 12 months of PFS or had progressive disease (PD) or stable disease (SD) were categorized as non-responders. Survival time was not used if the patient had no event and the follow-up time was censored before 12 months. Tumor samples obtained before induction of the therapy were termed “pre-treatment” samples, and tumors collected during or after the therapy were termed “on-treatment” samples.

### Linking gene expression and therapy response

Three separate analyses were performed across all genes to identify pre-treatment gene expression changes related to response against anti-PD-1, anti-PD-L1, and anti-CTLA-4 treatment. On-treatment samples were left out of the analysis because of low sample sizes.

We used Gene Ontology (GO) Enrichment analysis [38] to uncover biological processes connected to the gene lists related to response against anti-PD-1, anti-PD-L1, and anti-CTLA-4 treatment.

Druggability of candidate genes was determined by a literature search in PubMed and GeneCards (https://www.genecards.org/) to include those where (1) in silico prediction, (2) in vitro assay, (3) clinical study or (4) FDA-approval of the given drug were available.

### Validation of the results

We extended our previously established ROC plotter platform to enable the investigation of new biomarkers and the validation of current results in all patients treated with immunotherapy. The platform is running on Ubuntu 20.04.4 LTS server driven by Apache 2.4.41. The front-end site was developed in PHP using the YII2 framework. The application data are stored in the PostgreSQL database and the computations are performed via an R script. The portal can be reached at https://www.rocplot.com/immune.

### Statistical analysis

Statistical analysis was conducted in the R environment (https://www.r-project.org/) using Bioconductor libraries (https://www.bioconductor.org/). To find differentially expressed genes associated with improved or worse outcomes, Mann-Whitney unpaired U-test and receiver operating characteristic (ROC) analysis were used. To avoid false discovery due to multiple testing, Bonferroni-adjustment (*P* = 0.05) was applied with the service https://www.multipletesting.com/ [39].

## Results

### Screening results

We have identified 225 series files in NCBI GEO fulfilling the initial search criteria. Through literature research, we also found the Cancer Research Institute iAtlas (CRI iAtlas) (https://www.cri-iatlas.org/), another portal with ICI-treated samples [40], in which another six datasets were found. Finally, five additional cohorts were found by looking up the referenced literature [30, 37, 41–43].

**Fig. 1:**
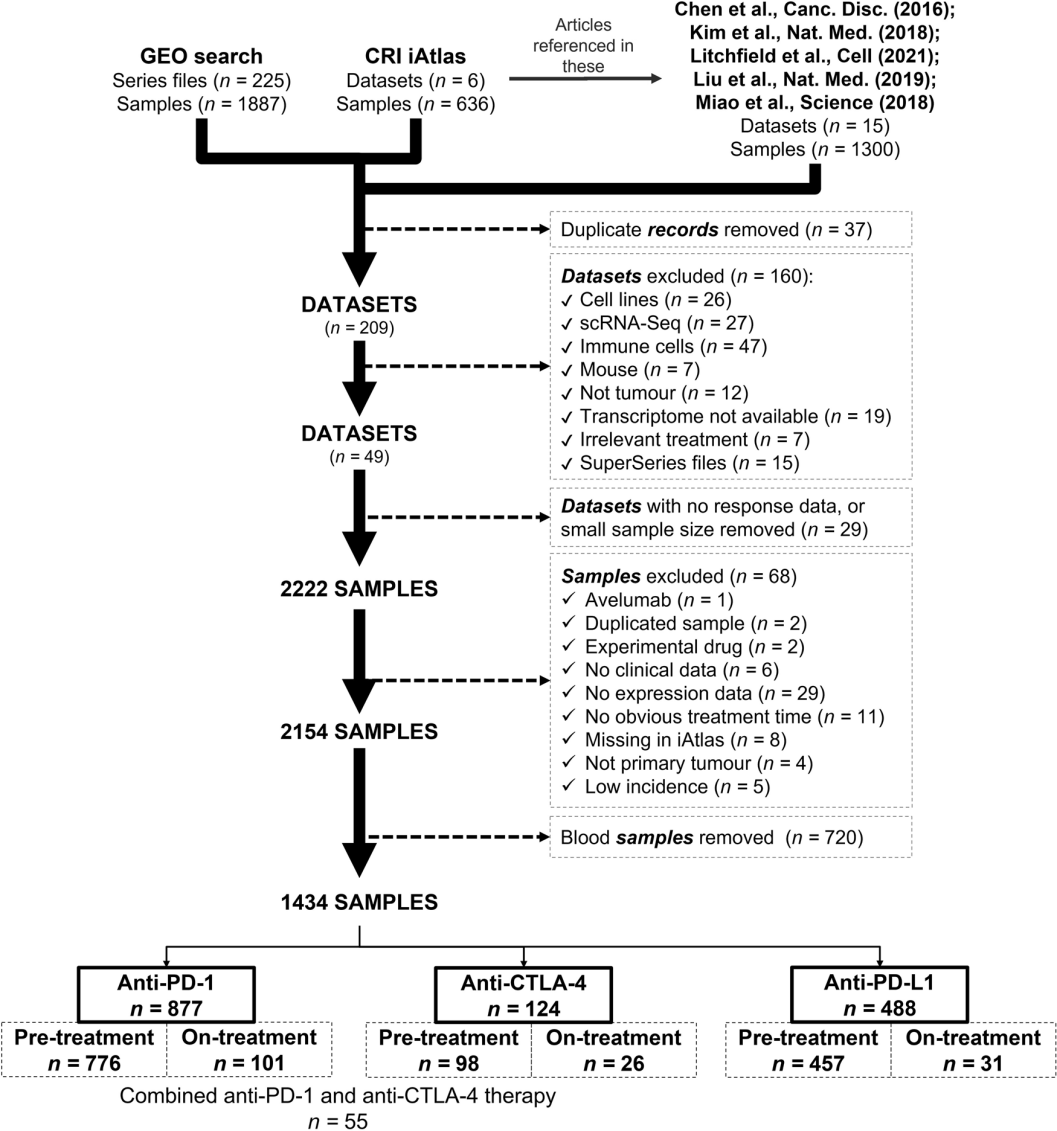
Screening datasets. Setup of the integrated database

In summary, from NCBI GEO [44, 45], CRI iAtlas, Chen et al. [43], Litchfield et al. [41], Liu et al. [37], Kim et al. [30], and Miao et al. [42], altogether 246 datasets with 3823 samples were found. Then, we removed duplicate records. For example, Litchfield et al. described eleven studies out of which four were also included in CRI iAtlas, and two in GEO (GSE78220, GSE91061). A detailed description of the complete screening process is provided in Fig. 1.

### Database assembly

Datasets were individually investigated to include only those in which at least one of the following clinical variables was reported: progression-free survival or interval (PFS/PFI), relapse-free survival (RFS), overall survival (OS), recurrence, and response determined by the Response Evaluation Criteria in Solid Tumors (RECIST) (including complete response (CR), partial response (PR), stable disease (SD), or progressive disease (PD)).

Twenty datasets with 2222 samples met these eligibility criteria and were selected for further analysis (Fig. 1). Sixty-eight samples were excluded due to different restrictions, such as (1) a very low number of samples treated with a specific agent (e.g., avelumab or experimental drugs), (2) duplicated samples, (3) missing clinical variable (e.g., event for survival), (4) no expression data, (5) ambiguous treatment time, (6) samples taken from metastatic sites or (7) low incidence of the given tumor type. By using these filtering criteria, the cohort was reduced to 2,154 samples out of which 720 samples were blood samples – these were removed from further analysis.

Finally, 1434 samples from 19 datasets were investigated manually. Different tumor types were integrated into the database, including (a) melanoma (*n* = 570), (b) esophageal and gastroesophageal junction adenocarcinoma (*n* = 103), (c) gastric cancer (*n* = 45), (d) urothelial cancer (bladder/ureter/pelvis cancer) (*n* = 449), (e) head and neck squamous cell carcinoma (*n* = 110), (f) hepatocellular carcinoma (*n* = 22), g) lung cancer (small cell and non-small-cell lung cancer, or squamous and non-squamous non-small cell lung cancer) (*n* = 60), (h) breast cancer (triple-negative (*n* = 12), and ER + HER2- breast cancer (*n* = 2)), (i) renal cell carcinoma (*n* = 33), and (j) glioblastoma (*n* = 28). Patients either received anti-PD-1 (nivolumab, or pembrolizumab) (all *n* = 877; pre-treatment *n* = 776; on-treatment *n* = 101), anti-PD-L1 (atezolizumab, or durvalumab) (all *n* = 488; pre-treatment *n* = 457; on-treatment *n* = 31), or anti-CTLA-4 (ipilimumab) (all *n* = 124; pre-treatment *n* = 98; on-treatment *n* = 26) treatments, out of which 55 patients received combination therapy with anti-PD-1 (Fig. 1).

Eventually, datasets were stratified into six groups: (1) pre-treatment anti-PD-1 (GSE78220, GSE91061, GSE93157, GSE115821, GSE121810, GSE136961, GSE140901, GSE165745, GSE176307, Cristescu2018, Gide2019, Kim2018, Miao2018, and Liu2019), (2) on-treatment anti-PD-1 (GSE78220, GSE91061, GSE115821, GSE121810, Gide2019, and Liu2019), (3) pre-treatment anti-PD-L1 (GSE165252, GSE176307, GSE183924, Mariathasan2018, and Miao2018), (4) on-treatment anti-PD-L1 (GSE165252), (5) pre-treatment anti-CTLA-4 (GSE115821, GSE140901, GSE165278, Gide2019, Miao2018, and VanAllen2015), and (6) on-treatment anti-CTLA-4 (GSE115821, GSE165278, and Gide2019) cohort. Due to the relatively small sample sizes, on-treatment datasets were excluded and three separate analyses were performed using the pre-treatment samples only. A summary of each dataset is listed in Table 1.

**Table 1:** Summary of cancer datasets with immune-checkpoint inhibitor therapy included in the study

Dataset ID	Reference	Sample *n*	Patient *n*	Tumor type	Drug	Sample acquisition	Outcome	Method for measuring gene expression
GSE78220	NCBI GEO and CRI iAtlas	28	27	Melanoma	Pembrolizumab	Pre-treatment	OS and response by RECIST	RNA-Seq (Illumina HiSeq 2000)
GSE91061	CRI iAtlas	98	56	Melanoma	Nivolumab	Pre-treatment or On-treatment	OS and response by RECIST	RNA-Seq (Illumina Genome Analyzer)
GSE93157	NCBI GEO	5	65	Head and Neck Squamous Cell Carcinoma	Nivolumab	Pre-treatment	PFS and response by RECIST	NanoString nCounter PanCancer Immune Profiling Panel
		22		Non-Squamous Non-Small Cell Lung Cancer	Nivolumab or Pembrolizumab	Pre-treatment	PFS and response by RECIST	
		25		Skin Cutaneous Melanoma	Nivolumab or Pembrolizumab	Pre-treatment	PFS and response by RECIST	
		13		Squamous Non-Small Cell Lung Cancer	Nivolumab or Pembrolizumab	Pre-treatment	PFS and response by RECIST	
GSE115821	NCBI GEO	37	11	Melanoma	Anti-PD-1 and/ or Anti-CTLA-4	Pre-treatment or On-treatment	Response	RNA-Seq (Illumina HiSeq 2000 and Illumina NextSeq 500)
GSE121810	NCBI GEO and CRI iAtlas	28	28	Glioblastoma	Pembrolizumab	Pre-treatment or On-treatment	PFI, OS, response by RECIST	RNA-Seq (Illumina HiSeq 3000)
GSE136961	NCBI GEO	21	21	Non-Small-Cell Lung Cancer	Anti-PD-1	Pre-treatment	PFS, OS	RNA-Seq (Ion Torrent S5 XL)
GSE140901	NCBI GEO	22	22	Hepatocellular Carcinoma	Nivolumab and/ or Ipilimumab	Pre-treatment	PFS, OS, response by RECIST	NanoString nCounter PanCancer Immune Profiling Panel
GSE165252	NCBI GEO and Provided by the author upon request	66	40	Esophageal Adenocarcinoma	Atezolizumab	Pre-treatment or On-treatment	PFS, OS, and response	RNA-Seq (Illumina HiSeq 4000)
GSE165278	NCBI GEO and GITHUB	21	21	Melanoma	Ipilimumab	Pre-treatment or On-treatment	OS	RNA-Seq (Illumina HiSeq 2500)
GSE165745	NCBI GEO	24	24	Melanoma	Pembrolizumab or Nivolumab	Pre-treatment	Response	NanoString nCounter Vantage 3D Human Wnt Pathways Panel
GSE176307	NCBI GEO	84	84	Bladder/ Ureter/ Pelvis Cancer	Pembrolizumab/ Nivolumab, or Atezolizumab/ Durvalumab	Pre-treatment	PFS and response by RECIST	RNA-Seq (Ion Torrent S5 XL)
GSE183924	NCBI GEO	37	37	Esophageal and Gastroesophageal Junction Adenocarcinoma	Durvalumab	Pre-treatment	RFS	RNA-Seq (Illumina NovaSeq 6000)
CRISTESCU 2018	Cristescu et al., Science (2018) and Litchfield et al., Cell (2021)	17	226	Bladder Cancer	Pembrolizumab	Pre-treatment	Response	NanoString nCounter
		12		Tripla Negative Breast Cancer	Pembrolizumab	Pre-treatment	Response	
		2		ER + HER2- Breast Cancer	Pembrolizumab	Pre-treatment	Response	
		105		Head and Neck Squamous Cell Carcinoma	Pembrolizumab	Pre-treatment	Response	
		86		Melanoma	Pembrolizumab	Pre-treatment	Response	
		4		Small Cell Lung Cancer	Pembrolizumab	Pre-treatment	Response	
GIDE 2019	CRI iAtlas	88	72	Melanoma	Pembrolizumab and/ or Nivolumab and/ or Ipilimumab	Pre-treatment	PFI, OS, response by RECIST	RNA-Seq (Illumina HiSeq 2500)
KIM 2018	ENA and Kim et al., Nat Med (2018)	45	45	Gastric Cancer	Pembrolizumab	Pre-treatment	Response by RECIST	RNA-Seq (Illumina NextSeq 550)
LIU 2019	Liu et al., Nat Med (2019)	121	121	Melanoma	Nivolumab or Pembrolizumab	Pre-treatment	PFS, OS, response by RECIST	RNA-Seq (Illumina HiSeq 2000 v3, HiSeq 2500)
MARIATHASAN 2018	CRI iAtlas	348	348	Urothelial Cancer	Atezolizumab	Pre-treatment	OS and response by RECIST	RNA-Seq (Illumina TruSeq RNA Access)
MIAO 2018	Miao et al., Science (2018)	33	33	Renal Cell Carcinoma	Nivolumab and/ or Ipilimumab, or Atezolizumab	Pre-treatment	PFS, OS, and response by RECIST	RNA-Seq (Illumina HiSeq 2000 v3, HiSeq 2500)
VANALLEN 2015	CRI iAtlas and Van Allen et al., Science (2015)	42	42	Melanoma	Ipilimumab	Pre-treatment	PFS, OS, and response by RECIST	RNA-Seq (Illumina HiSeq 2500)

Different technological, and clinical approaches were utilized in the involved studies such as RNA-sequencing (RNA-Seq) with Illumina [46] or Ion Torrent platforms [47], and NanoString nCounter [48, 49]. Regarding therapies, many studies used combination therapy e.g., anti-PD-1, or anti-PD-L1, with or without anti-CTLA-4 therapy [42], or anti-PD-1 monotherapy, with or without anti-CTLA-4 treatment [50, 51], while others investigated monotherapies only – such as ipilimumab [52], atezolizumab [53], or durvalumab [54].

### Druggable genes with higher expression related to resistance to anti-PD1 administration

**Fig. 2:**
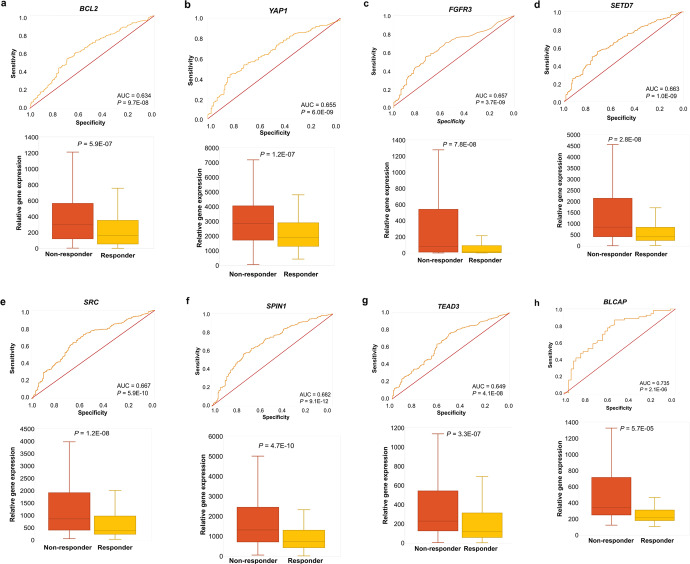
Top genes of anti-PD-1, or anti-CTLA-4 resistance. ROC-plots and boxplots of best druggable candidate genes predicting resistance in anti-PD-1 pre-treatment (*BCL2* (**a**), *YAP1* (**b**), *FGFR3* (**c**) *SETD7* (**d**) *SRC* (**e**), *SPIN1* (**f**), and *TEAD3* (**g**)), and anti-CTLA-4 pre-treatment groups (*BLCAP* (**h**)).

First, the pre-treatment samples in group #1 were investigated by computing ROC AUC and *P*-values for 29,755 genes. Following Bonferroni correction, values reaching more than *P* = 1.6E-06 were excluded from further analysis, which led us to 912 significant genes. The most significant hits included *MARCKS* (FC = 2.2, AUC = 0.724, *P* = 4.2E-12), *SPIN1* (FC = 1.6, AUC = 0.682, *P* = 9.1E-12), *PAK5* (FC = 1.8, AUC = 0.677, *P* = 6.1E-08), *DDAH1* (FC = 1.7, AUC = 0.671, *P* = 3.4E-10), *ELOVL6* (FC = 2.1, AUC = 0.667, *P* = 3.5E-10), *SRC* (FC = 1.6, AUC = 0.667, *P* = 5.9E-10), *SETD7* (FC = 1.7, AUC = 0.663, *P* = 1.0E-09), *FGFR3* (FC = 2.1, AUC = 0.657, *P* = 3.7E-09), *YAP1* (FC = 1.6, AUC = 0.655, *P* = 6.0E-09), *BACE1* (FC = 1.6, AUC = 0.654, *P* = 7.8E-09), *STK35* (FC = 1.7, AUC = 0.651, *P* = 1.4E-08), *TEAD3* (FC = 1.7, AUC = 0.649, *P* = 4.1E-08), *TMPRSS4* (FC = 1.5, AUC = 0.640, *P* = 2.5E-07), *PP1CB* (FC = 1.6, AUC = 0.639, *P* = 2.0E-07), and *BCL2* (FC = 2.2, AUC = 0.634, *P* = 9.7E-08) which were all upregulated in the non-responder group (Fig. 2). The complete list of all significant genes is provided in Supplementary Table S1. Notably, non-protein coding genes (such as pseudogenes, long intergenic non-protein coding RNAs, antisense RNAs, regulatory RNAs, small nucleolar RNAs, open reading frame, etc.) were excluded from our screening.

By using all significant genes (*n* = 912) for GO analysis, mechanisms such as retrograde transport, vesicle recycling within Golgi (GO:0000301), ncRNA catabolic process (GO:0034661), and T cell receptor signaling pathway (GO:0050852) were significantly overrepresented among these genes (Supplementary Table S2).

### Druggable genes with higher expression related to resistance to anti-PD-L1 treatment

ROC AUC and *P*-values from 26,819 genes were computed and following Bonferroni-correction, values over *P* = 1.8E−06 were excluded. This way, we identified 38 significant genes. The complete list of all significant genes can be found in Supplementary Table S3. The Gene Ontology analysis shows that mechanisms connected to the C-type lectin receptor signaling pathway (GO:0002223), cellular response to lectin (GO:1990858), and positive regulation of natural killer cell-mediated cytotoxicity (GO:0045954) were overrepresented in the list of significant genes (*n* = 38) (Supplementary Table S2). There were no upregulated, druggable genes capable to predict resistance against anti-PD-L1 therapy.

### Druggable genes with higher expression related to anti-CTLA-4 treatment resistance

In this third analysis, ROC AUC and *P*-values were calculated for 22,561 genes in the pre-treatment group. Of these, 80 genes reached significance after Bonferroni correction. Among them, *BLCAP* (FC = 1.7, AUC = 0.735, *P* = 2.1E−06) was found as a druggable gene overexpressed amongst non-responding patients (Fig. 2). The complete gene list can be found in Supplementary Table S4. Non-protein coding genes were also excluded from this group. The GO analysis with multiple testing correction delivered no significant classification for these genes.

### Established cancer biomarkers and response to immunotherapy

We also investigated the power of established cancer biomarkers for predicting therapeutic response to immune checkpoint inhibitors. For this, a previously described panel of cancer biomarkers was utilized [55] (see the complete list with results in Supplementary Table S5). The analysis was performed with two cohorts including pre-treatment anti-PD-1, and pre-treatment anti-CTLA-4 samples. Following Bonferroni-correction, only one gene, *ALK* (Anaplastic Lymphoma Receptor Tyrosine Kinase, FC = 1.6, AUC = 0.612, *P* = 3.3E−05) showed a correlation with anti-PD-1 response. In case of anti-CTLA-4, *CD19* (FC = 1.6, AUC = 0.666, *P* = 6.5E−04) and *PGR* (Progesterone Receptor, FC = 1.2, AUC = 0.639, *P* = 6.8E−03) were found to be predictive. Notably, *CD274* (PD-L1), as expected, was overexpressed in responding patients, however, failed to pass the significance threshold after Bonferroni-correction (*P* = 1.6E−06).

### Mismatch-repair genes and response against anti-PD-1 treatment

**Fig. 3:**
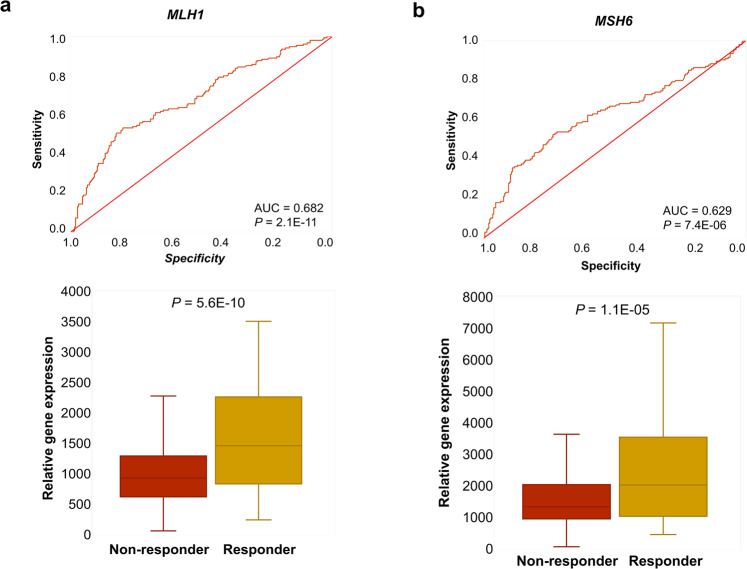
MLH1, and MSH6 in the anti-PD-1 pre-treatment group. ROC-plots for predicting sensitivity to anti-PD-1 treatment, and boxplots of gene expression comparing responder and non-responder samples for *MLH1* (**a**) and *MSH6* (**b**) in a combined dataset of anti-PD-1 pre-treatment samples.

Finally, we aimed to determine to what extent one can predict sensitivity to the anti-PD-1 dostarlimab using the integrated database of published datasets. For this, we performed ROC analysis to evaluate the anti-PD-1 biomarkers previously published in a cohort of rectal cancer [56]. We used transcriptomic data of 419 samples from melanoma, bladder, renal cell, and gastric cancer in the anti-PD-1 pre-treatment cohort (*n* = 776). In this analysis, *MLH1* and *MSH6* achieved high predictive values (FC = 1.5, AUC = 0.682, *P* = 2.1E−11 and FC = 1.4, AUC = 0.629, *P* = 7.4E−06, respectively). Notably, 218 genes reached even higher ROC AUC than *MLH1* (Fig. 3).

## Discussion

Here, we integrated available data from multiple datasets and used this combined database to uncover biomarkers related to response against ICIs in three independent clinical settings. An advantage of the presented analysis pipeline is the utilization of real-world patient data. While most of the individual studies have only a limited number of samples, our combined patient cohort with well over a thousand patients provides a higher statistical power.

Among the most significant genes related to resistance against anti-PD-1 treatment, we identified several potentially druggable targets. Fibroblast Growth Factor Receptor 3 (FGFR3) plays a pivotal role in tumorigenesis and the regulation of innate and adaptive immune systems [57]. Overexpression of FGFR3 is associated with an immunologically cold, T cell-depleted phenotype, which contributes to a low ICI response rate in bladder cancer [58]—just like a low PD-L1 expression in an FGFR3-mutant scenario [59]. From multikinase inhibitors (such as anlotinib, dovitinib, lenvatinib, and ponatinib) to selective FGFR inhibitors (e.g., erdafitinib, infigratinib, pemigatinib), various small molecule inhibitors are available for solid tumors and lymphohematopoietic cancers [60, 61]. The combination of FGFR and PD-1 inhibition might also be beneficial [62]. The Src Proto-Oncogene Non-Receptor Tyrosine Kinase (SRC) is a well-known oncogene contributing to cell growth, cell proliferation, and survival. Tumor-induced cytokines, e.g., Macrophage Inflammatory Protein 1, and 2 (MIP-1 and MIP-2) activate Src kinases in immune cells which lead to the production of pro-inflammatory cytokines (e.g., interleukin-1ß and 6, Tumor Necrosis Factor α) that activate cancer cells in a positive feedback loop [63]. Multikinase inhibitors, such as the FDA-approved bosutinib, dasatinib, ponatinib, and vandetanib are currently being used for the treatment of chronic myeloid leukemia, acute lymphoblastic leukemia, and patients with thyroid cancer [64]. B-Cell Lymphoma 2 (BCL2) is a major regulator of the “apoptotic-orchestra” by inhibiting apoptosis and promoting cell survival [65]. Immune checkpoint molecules themselves promote an anti-apoptotic phenotype—leading to immune tolerance and low response rates [66]. Venetoclax is the only FDA-approved small-molecule inhibitor against BCL2 in acute myeloid leukemia (AML), and chronic lymphatic leukemia—yet other drugs might follow both in hematologic [67] and solid tumor malignancies [68]. Yes-Associated Protein 1 (YAP1) is a transcriptional coactivator, which upon binding to many transcription factors, such as TEAD3 (Transcriptional Enhanced Associate Domain 3), regulates the Hippo-signalling pathway, contributing to tumor growth, and resistance [69]. The Hippo-YAP system interferes with the innate immune system in multiple ways such as inhibiting the production of type I interferons (IFN-α, IFN-ß) and reactive oxygen species (ROS), attenuating NF-κB activation, or enhancing TNF-α and IL-1ß production [70]. These events contribute to the suppression of the innate immune system thus escaping immune recognition – which eventually leads to tumor survival. Verteporfin (VP) is widely used for the treatment of macular degeneration, however, current studies highlighted the antitumor effects of VP either with photoactivation or without it [71]. The capability of CA3, Super-TDU, statins, sitagliptin, SRC, FAK (Focal Adhesion Kinase), and tankyrase inhibitors to disrupt the YAP-TEAD complex were also discussed previously [72]. SET Domain Containing Lysine Methyltransferase 7 (SETD7) has a broad target-specificity as it is involved in many biological processes by interacting with p53, Estrogen Receptor-Alpha (ERα), or YAP1. For this reason, SETD7 can both activate and inhibit tumor-survival signals [73, 74]. Upon methylation on K494 by SETD7, YAP1 accumulates in the cytoplasm and blocks the Hippo-pathway [75]. This leads to the nuclear accumulation of ß-catenin and the activation of the Wnt/ß-catenin pathway which is one of the most well-known oncogenic pathways [76]. Besides having a direct effect on cell proliferation and stemness, ß-catenin promotes a non-inflammatory milieu in tumors by inhibiting the activation and recruitment of CD8^+^ T cells and enhancing the infiltration and survival of regulatory T cells (Tregs). This leads to resistance to ICIs, emphasizing the potential of Wnt/ß-catenin as a predictive biomarker, or as a therapeutic target [77]. Moreover, SETD7 can methylate p65 and thereby inhibit the expression of NF-κB, and is a positive regulator of Transforming Growth Factor Beta (TGF-ß) production – all these contribute to tumorigenesis [78]. There are some promising results of inhibiting SETD7 with DC-S100 [79], DC-S285 [80], cyproheptadine [81], and ®-PFI-2 [73]. Spindlin 1 (SPIN1) is a histone methylation reader contributing to the epigenetic regulation of many oncogenic pathways so it is not surprising that SPIN1 was found to be overexpressed in many cancers [82]. Notably, SPIN1 acts as a transcriptional coactivator of ß-catenin and T cell Factor 4 (TCF-4) enhancing their contribution to Wnt/TCF-4 pathways, which leads to tumor progression [83]. Inhibitors of SPIN1 are being studied—e.g., A366, EML405, MS31, 4-aminoquinazoline and quinazolinethione derivatives, or VinSpinIn [82].

We have found only one gene related to anti-CTLA-4 resistance: BLCAP or Bladder Cancer Associated Protein. As an apoptosis-inducing factor, BLCAP can initiate apoptosis in many tumors and is considered a tumor suppressor gene. Lost expression or degradation of BLCAP is observed in urothelial, renal, and cervical cancer, osteosarcoma, colorectal carcinoma, and human tongue carcinoma [84, 85]. Nonetheless, poor survival of bladder cancer patients correlates with strong nuclear expression of BLCAP [86], which is impacted by the interaction of BLCAP and Signal Transducer and Activator of Transcription 3 (STAT3) in the JAK/STAT-pathway [85]. Selective pharmacological inhibition of BLCAP can be observed with aristolochic acid in vitro [87].

In locally advanced rectal cancer, the loss of mismatch-repair genes was a highly significant biomarker of response to the anti-PD-1 inhibitor dostarlimab [56]. When re-evaluating the previously published genes, we were able to confirm a significant correlation with survival in our cohort for *MLH1* and *MSH6*. Nevertheless, 218 genes reached even higher significance than MLH1 in these patients, pointing out that other biomarkers might be even more suitable to select patient cohorts for immunotherapy. The samples in our patient cohort stemmed from melanoma, bladder, renal cell, and gastric cancer suggesting that the loss of mismatch-repair genes could also enhance sensitivity to anti-PD-1 therapy in these tumor types.

There are some limitations of our study. Despite the importance of this topic, only a relatively small number of datasets have been found and included in the analysis. Since the currently used drugs are targeting proteins, an addition of protein-level data would have been useful. Unfortunately, we have not found even one dataset with additional protein abundance data. Finally, as ICIs are approved for advanced cancers, most patients have already received multiple treatment regimes, which might have resulted in significant background noise at the transcriptomic level preventing the identification of the most reliable biomarker candidates.

In summary, we have established a database consisting of gene expression and clinical response data by combining 1434 solid tumor tissue samples obtained before or after immune-checkpoint inhibitor treatment. The most significantly upregulated, pharmacologically important (druggable) genes were identified in connection with the resistance against anti-PD-1, and anti-CTLA-4 treatments. Our extended analysis platform can help to identify, validate, and rank future biomarker candidates.

## Supplementary information

Supplementary Table S1

Supplementary Table S2

Supplementary Table S3

Supplementary Table S4

Supplementary Table S5

Supplementary Information

## Data Availability

The original data used in the publications are available from GEO or CRI iAtlas using their respective identifiers, or accessible via the indicated publications.
