# Supplementary material for: Predictive biomarkers of immunotherapy response with pharmacological applications in solid tumors
Source: Acta Pharmacol Sin. 2023 Apr 13;44(9):1879–89. doi: 10.1038/s41401-023-01079-6 (PMC10462766; doi:10.1038/s41401-023-01079-6)
Supplement: Supplementary file 6 — Supplementary Information [file 41401_2023_1079_MOESM6_ESM.docx]

# SUPPLEMENTARY INFORMATION

**Supplementary Table S1.** Results of Mann-Whitney and ROC-analysis across all genes in the anti-PD-1 treatment group.

**Supplementary Table S2.** Results of Gene Ontology analysis in all treatment groups.

**Supplementary Table S3.** Results of Mann-Whitney and ROC-analysis across all genes in the anti-PD-L1 treatment group.

**Supplementary Table S4.** Results of Mann-Whitney and ROC-analysis across all genes in the anti-CTLA-4 treatment group.

**Supplementary Table S5.** Results of Mann-Whitney and ROC-analysis for established cancer biomarker genes.
